# Deep learning models for tendinopathy detection: a systematic review and meta-analysis of diagnostic tests

**DOI:** 10.1530/EOR-24-0016

**Published:** 2024-10-03

**Authors:** Guillermo Droppelmann, Constanza Rodríguez, Dali Smague, Carlos Jorquera, Felipe Feijoo

**Affiliations:** 1 Research Center on Medicine, Exercise, Sport and Health, MEDS Clinic, Santiago, RM, Chile; 2 Health Sciences PhD Program, Universidad Católica de Murcia UCAM, Murcia, Spain; 3 Harvard T.H. Chan School of Public Health, Boston, USA; 4 Facultad de Medicina, Universidad Finis Terrae, Santiago, RM, Chile; 5 Facultad de Ciencias, Escuela de Nutrición y Dietética, Universidad Mayor, Santiago, RM, Chile; 6 School of Industrial Engineering, Pontificia Universidad Católica de Valparaíso, Valparaíso, Chile

**Keywords:** artificial intelligence, deep learning, diagnostic, orthopedics, radiology, tendinopathy

## Abstract

**Purpose:**

- Different deep-learning models have been employed to aid in the diagnosis of musculoskeletal pathologies. The diagnosis of tendon pathologies could particularly benefit from applying these technologies. The objective of this study is to assess the performance of deep learning models in diagnosing tendon pathologies using various imaging modalities.

**Methods:**

- A meta-analysis was conducted, with searches performed on MEDLINE/PubMed, SCOPUS, Cochrane Library, Lilacs, and SciELO. The QUADAS-2 tool was employed to assess the quality of the studies. Diagnostic measures, such as sensitivity, specificity, diagnostic odds ratio, positive and negative likelihood ratios, area under the curve, and summary receiver operating characteristic, were included using a random-effects model. Heterogeneity and subgroup analyses were also conducted. All statistical analyses and plots were generated using the R software package. The PROSPERO ID is CRD42024506491.

**Results:**

- Eleven deep-learning models from six articles were analyzed. In the random effects models, the sensitivity and specificity of the algorithms for detecting tendon conditions were 0.910 (95% CI: 0.865; 0.940) and 0.954 (0.909; 0.977). The PLR, NLR, lnDOR, and AUC estimates were found to be 37.075 (95%CI: 4.654; 69.496), 0.114 (95%CI: 0.056; 0.171), 5.160 (95% CI: 4.070; 6.250) with a (*P* < 0.001), and 96%, respectively.

**Conclusion:**

- The deep-learning algorithms demonstrated a high level of accuracy level in detecting tendon anomalies. The overall robust performance suggests their potential application as a valuable complementary tool in diagnosing medical images.

## Introduction

Mechanization, electrification, and automation drove the first three industrial revolutions, gradually transforming the manufacturing-based way of life. These revolutions had a positive impact on the quality of life, including the healthcare system of the population (1). The current Fourth Industrial Revolution (4IR, or Industry 4.0) is a combination of multiple digital and software technologies. Artificial intelligence (AI) stands out as the main engine of this industry, empowering computers with models and algorithms to solve problems, make decisions, and simulate activities inherent to human beings (2).

The application of AI has shown enormous potential in tasks such as predictive analysis, inventory management, supply chain management, industrial robotics, and computer vision. The latter, due to its vast scope and significant transformative capacity in healthcare practices, has taken a predominant position (3). Process optimization and continuous improvement in patient monitoring have enhanced the accuracy of diagnosis. Its ability to autonomously learn and recognize complex patterns, as well as analyze and segment potential anomalies in various medical images such as magnetic resonance imaging, X-rays, computed tomography, and ultrasounds, makes it applicable in radiological diagnosis (4).

At the same time, various AI-driven algorithms lead these activities, among which k-Nearest Neighbors (k-NN), Naive Bayes, Artificial Neural Networks (ANN), and Deep Learning (DL) stand out. These algorithms significantly reduce the time spent on repetitive activities, decrease the maintenance cost of technological equipment, and improve various diagnostic processes (5). Each of the algorithms has strengths but also certain weaknesses that could impact accuracy, speed, and robustness (1). For this reason, new alternatives are proposed every day to address the emerging challenges posed by AI-powered radiology (6).

In the field of image recognition, DL has been considered the gold standard within the machine learning (ML) community, becoming the most widely used computational approach in this field. The way it achieves outstanding results in complex cognitive tasks has allowed it to match or even surpass the performance of activities carried out by trained humans (7). Methods ranging from convolutional neural networks (CNN) to variational autoencoders have found countless applications in the field of medical image analysis, propelling it forward at a rapid pace (8). Among the main strengths of DL, its ability to capture features from a radiological image without human intervention stands out directly. It possesses wide flexibility for manipulation, high diagnostic precision, significant processing capability, and real-time adaptability. Notably, it also highlights its indirect impact by enhancing the performance of professionals and their hospital environment, reducing diagnostic uncertainty in decision-making. The decrease in the workload of the medical specialist team alleviates waiting lists, thus improving the management and efficiency of radiology services (9).

DL architectures have been implemented for several decades. Recently, DL techniques for image recognition have piqued the interest of the radiological community due to their superior diagnostic accuracy compared to ML (4). For example, the first recorded architecture was a Shallow Neural Network in 1940, followed by K-means in 1960, Multilayer Neural Network, and Backpropagation Algorithm in 1960–1970, Neocognition in 1979, Decision Trees and Bayesian Network in 1980, Convolutional Neural Network (CNN) in 1989, Super Vector Machine (SVM), and Clustering in 1990. The most popular ones for these purposes include VGG, created in 2003; Inception-V1 in 2006; ResNet-50 in 2011; and AlexNet in 2012. Currently, researchers have proposed hybrid models, as well as more sophisticated models compared to the original proposals, such as Inception ResNets from 2017 or ResNet-18 from 2019 (10).

Particularly, DL stands out, especially among other types of learning, due to its ability to learn representations from raw data. This is because the architecture of DL consists of multiple layers of information processing based on the hierarchical structures of neural networks. These networks learn data representations with various levels of abstraction (11). In other words, the complexity of a deep neural network will depend on the number of hidden layers, their connections, and the ability to learn meaningful abstractions from the inputs (12).

**Figure 1:**
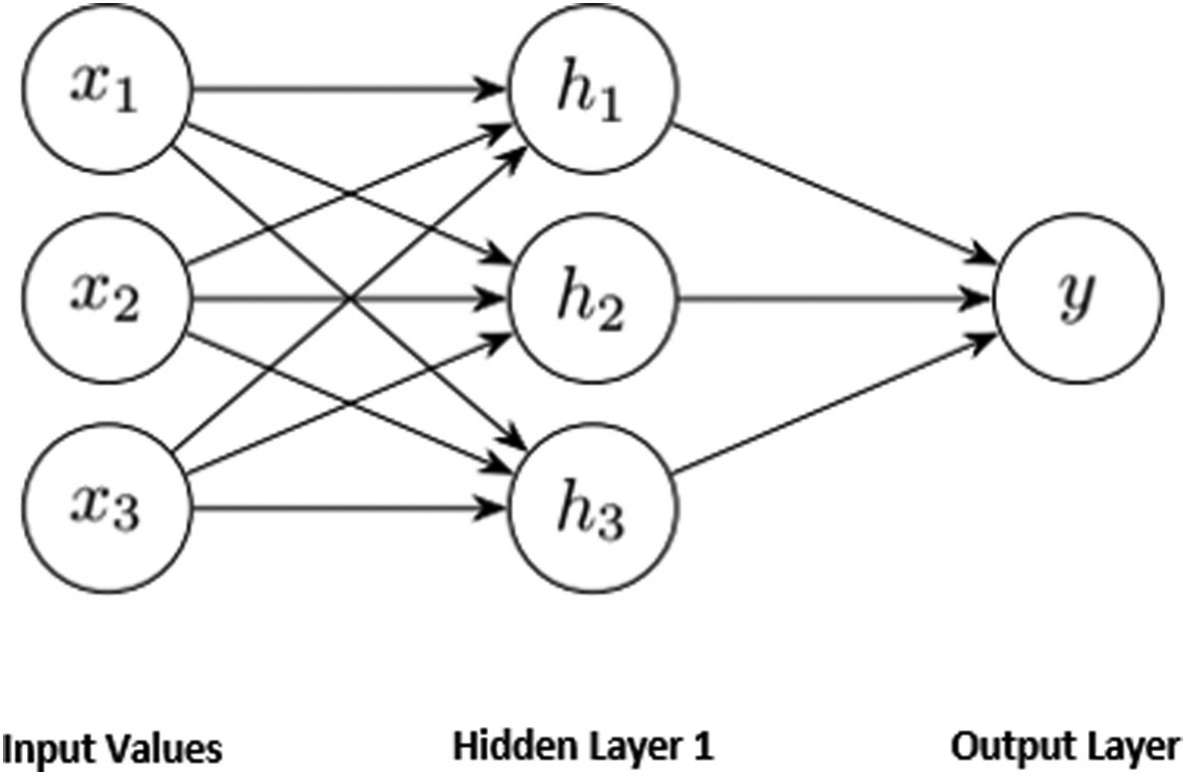
Deep learning architecture.

Each layer of a deep learning system generates a representation of observed patterns by optimizing a local unsupervised criterion (13). This is in contrast to traditional ANNs, which often have limitations with three layers and are designed to obtain supervised representations optimized solely for specific tasks. Therefore, it is expected that deep learning systems with a greater number of layers will exhibit better performance than those with fewer layers. However, adding more layers does not automatically guarantee improved performance in all cases. Factors such as representational capacity, gradients of the layers, computational resources, hyperparameters, training time, overfitting, and the nature of the data according to the model architecture should be carefully considered (14, 15). Figure 1 illustrates the organization of the general structure of a deep neural network with one hidden layer.

Recently, various publications and systematic literature reviews have identified the pathologies that have mostly benefited from the use of these diagnostic methods. For example, breast disease (21%), brain tumors (18%), diabetes (16%), and lung diseases (16%), as well as conditions affecting the eyes (16), liver (17), and skin (18). Particularly, there are architectures that stand out for their excellent performance in image processing (19). Among them is the VGG16, and as its name suggests, it has 16 layers, which, due to not having a large number of hyperparameters, demonstrates high performance in image classification tasks (20). InceptionV3 excels in computational efficiency due to its architecture evolving over time through factorized convolutions, reducing the number of network parameters and enhancing overall computer performance (21). Another noteworthy architecture is DenseNet129, an abbreviation for Dense Convolutional Network. This compact network optimizes resource utilization by employing fewer channels and reusing functions through a concatenation process (22). Lastly, ResNet50 stands out for its residual network design. The term “Residual” in ResNet signifies the incorporation of skip connections, providing an alternative path for the gradient to flow through the network. This innovation ensures that each level functions as proficiently as the preceding layer (23).

In this scenario, it is striking that the utilization of AI in diagnostic imaging for traumatology, orthopedics, and sports medicine remains relatively limited compared to its application in other medical fields that leverage these tools to enhance diagnostic capabilities. Primary efforts have focused on addressing conditions that affect the spine, identifying fractures, and detecting soft tissue abnormalities, including meniscal injuries in the knees (24). Furthermore, AI has been utilized in various areas including prosthesis control, gait classification, and the detection of osteoarthritis (25). Nevertheless, one musculoskeletal condition that exhibits a high prevalence and affects people worldwide is tendinopathy.

This tendon injury predominantly affects individuals in middle age who engage in moderate- to high-intensity physical activities or those who have undergone repetitive traumas over time (26). These factors present a considerable challenge to public health, exerting a significant impact on aging, quality of life, individual well-being, and the healthcare systems of countries. Therefore, having diagnostic strategies incorporating sophisticated algorithms from deep learning would not only keep abreast of other disciplines but also enhance various processes and decision-making in fields like musculoskeletal radiology, where precise diagnoses are paramount (27).

To the best knowledge of the authors, as of now, there is no scientific article that has explored models and DL algorithms for supporting the diagnosis of tendinopathies using any type of radiological imaging modality. The objective of this meta-analysis is to assess the diagnostic capability of deep learning algorithms and neural networks in identifying tendinopathies through various imaging examination modalities.

## Methods

### Reporting

This meta-analysis was conducted in accordance with the recommendations outlined in the Preferred Reporting Items for Systematic Reviews and Meta-Analyses (PRISMA) guidelines. The PRISMA 2020 statement provides a 27-item checklist covering the introduction, methods, results, and discussion sections of a systematic review report. The checklist is accessible at www.prisma-statement.org. The authors officially registered the review on the PROSPERO platform (ID: CRD42024506491).

### Research question

The primary research question addressed in this article is to evaluate the diagnostic accuracy of deep learning models and neural networks in identifying tendinopathies across various medical imaging modalities. The PICOT criteria (Participants, Interventions, Comparison, Outcome, and Time) are detailed in Table 1. Additionally, the authors sought to explore potential variations in diagnostic accuracy based on the type of architecture employed in the diverse studies under evaluation.

**Table 1:** PICOT strategy for this study.

PICOT acronym	PICOT component	PICOT component explanation
(P)	Population	Patients with imaging diagnosis of tendinopathy
(I)	Intervention	Diagnosis of tendinopathy using any deep learning model or neural network
(C)	Comparison	Model architecture
(O)	Outcome	Diagnostic performance using any deep learning model or neural network
(T)	Type of study	Diagnostic study

### Search strategy and data sources

Two authors, DS and CR, conducted an information search in various databases, including MEDLINE/PubMed (https://www.ncbi.nlm.nih.gov/pubmed/), SCOPUS (https://www.scopus.com/home.uri), Cochrane Library (https://www.cochranelibrary.com/), Lilacs (https://lilacs.bvsalud.org/en/), and SciELO (https://scielo.conicyt.cl/). Any discrepancies among the assigned reviewers were resolved by a third reviewer, CJ. The considered timeframe spanned 10 years, specifically from January 2013 to September 2023. Another author, GD, specializing in musculoskeletal injuries with over 15 years of experience, performed the selection of keywords related to the tendon concept. Validation of keywords related to the diagnostic concept was carried out by an external collaborating radiologist with over 12 years of experience in musculoskeletal pathologies.

Keywords related to the artificial intelligence concept were identified by FF, a PhD in engineering with over 15 years of experience. The PubMed search engine confirmed all concepts as Medical Subject Headings (MeSH) terms. Finally, the terms considered in this review were ‘tendon’, ‘tendinopathy’, ‘diagnosis’, ‘diagnostic imaging’, ‘deep learning’, ‘neural network’, ‘convolutional neural network’, and ‘artificial neural network’. A data matrix was created by utilizing all possible combinations of the defined words. We obtained access to all articles selected for this purpose.

### Selection criteria

The following inclusion criteria were considered: i) complete, published original scientific articles; ii) scientific articles focused on tendon as a study condition; iii) original scientific articles containing any type of radiological image without discriminating the lesion segment; iv) original scientific articles incorporating one or more models and/or algorithms from DL as a complementary diagnostic method; v) original scientific articles explicitly reporting true positive (TP), false positive (FP), true negative (TN), and false negative (FN) values to calculate sensitivity and specificity indicators; vi) original scientific articles published in English, Spanish, or Portuguese; vii) original scientific articles published within a timeframe not exceeding 10 years until September 2023.

The following exclusion criteria were applied: i) scientific articles such as review articles, letters, congress reports, papers, cadaveric articles, and technique descriptions; ii) medical or technological devices, sensors, virtual reality, or any type of tangible (hardware) or intangible (software) objects that do not utilize artificial intelligence algorithms.

### Data extraction

Two co-authors, CR and DS, independently conducted information extraction, with any discrepancies resolved by a third author, GD. Initially, scrutiny was applied to titles and abstracts, followed by the selection of complete original scientific articles that aligned with the established criteria. Duplicate manuscripts were systematically removed, and a data matrix was meticulously crafted using Microsoft Excel. From the chosen scientific articles, pertinent details such as authors and year of publication, country of origin, number of images, type of imaging, tendon condition, and type of algorithm were extracted.

In this meta-analysis, a granular approach was taken, individually considering records of true positives, false negatives, false positives, and true negatives (TP, FN, FP, and TN) for each model and algorithm reported in the selected articles. For instance, if an article presented findings for two models, each set of metrics was distinctly accounted for and denoted as Model A and Model B, and so forth. This methodological choice was made to comprehensively understand the specific performance of the identified models. Additionally, reported information on accuracy and area under the curve (AUC) values was meticulously documented.

### Ethical approval

All selected research adhered to the principles outlined in the Helsinki Declaration and received approval from a scientific ethics committee. Each study affirmed having obtained informed consent as appropriate.

### Risk of bias (Quality) assessment

The Quality Assessment of Diagnostic Accuracy Studies (QUADAS-2) guidelines (28) were employed as the tool for evaluating potential biases in the selected articles and assessing their overall quality. The evaluation encompasses three categories: low, unclear, or high, and involves the analysis of the following elements:

A) Risk of Bias: patient selection, index test, reference standard, and flow and timing. B) Applicability Concerns: patient selection, index test, and reference standard.

Researchers scrutinized the selection of patients for the study. The description of the index test provides specific details, covering its administration and interpretation. A comprehensive explanation is offered for the reference standard, elucidating its conduct and interpretation. The flow and timing section clarifies whether any patients did not undergo the index test or reference standard, establishing the time interval and any interventions between both assessments.

### Statistical analysis

Summary statistics, incorporating metrics such as TP (true positives), FN (false negatives), FP (false positives), and TN (true negatives), were calculated to capture the diagnostic accuracy of the tests. Univariate and bivariate analyses were performed for each deep learning (DL) model or neural network algorithm, following widely accepted guidelines for conducting meta-analyses (29, 30).

### Univariate analysis

Diagnostic accuracy was determined by considering both the number of events and the sample size in proportion-type data. Sensitivity and specificity were calculated individually and collectively for each model. Additionally, positive likelihood ratios (PLR) and negative likelihood ratios (NLR) were computed, along with their respective 95% CI. To ensure more stable results, a logistic transformation followed by an inverse transformation (Clopper-Pearson method) was applied. For ease of comparison and result aggregation, the pooled effect was estimated through the calculation of a diagnostic odds ratio (DOR) and its logarithmic transformation (lnDOR). Forest plots were employed for the graphical representation of the entire dataset.

### Bivariate analysis

A subgroup analysis was undertaken to address the secondary research question. The algorithms were arbitrarily classified into two groups (g) based on their architectural complexity. Group 0 (g = 0), characterized by low complexity, included algorithms such as VGG16, Xception, nnU-Net, ResNet, and VGG19. Meanwhile, group 1 (g = 1) encompassed models with higher complexity, including DenseNet, ATASM, AlexNet, CNN-2, ResNet50, and Inception-V3.

Diagnostic odds ratios were computed for each subgroup, and the results were visually presented using a forest plot. The researchers determined summary metrics for diagnostic accuracy using the AUC curve estimator and generated a Summary Receiver Operating Characteristic (SROC) curve.

### Heterogeneity analysis

A random-effects model was chosen due to the observed heterogeneity among the selected articles. Variability was calculated using the inverse variance method, considering the individual weights assigned to each study. Additionally, the DerSimonian-Laird estimator (tau value) was employed to estimate variability. The proportion of variability associated with heterogeneity, in contrast to random variability among studies, was assessed using Higgins’ *I*² indicator, both for the overall dataset and within subgroups. Values between 0% and 40% were considered indicative of minimal heterogeneity, while values between 30% and 60% suggested moderate heterogeneity. Values between 50% and 90% indicated high heterogeneity, and values between 75% and 100% demonstrated extreme heterogeneity. Cochran’s Q test was applied to calculate the total variability fraction attributable to differences in the sample.

### Packages and reports

To perform diagnostic accuracy analyses, the following packages were employed in the R statistical environment: ‘ellipse’, ‘mada’, ‘meta’, ‘metafor’, ‘mvmeta’, ‘mvtnorm’, and ‘rmeta’. A significance level of < 0.05 was set, and 95% CIs were computed. The results were reported with three decimal places. All statistical analyses and graphical representations were executed using the R statistical software package (version 4.1.3).

## Results

### Search results

**Figure 2:**
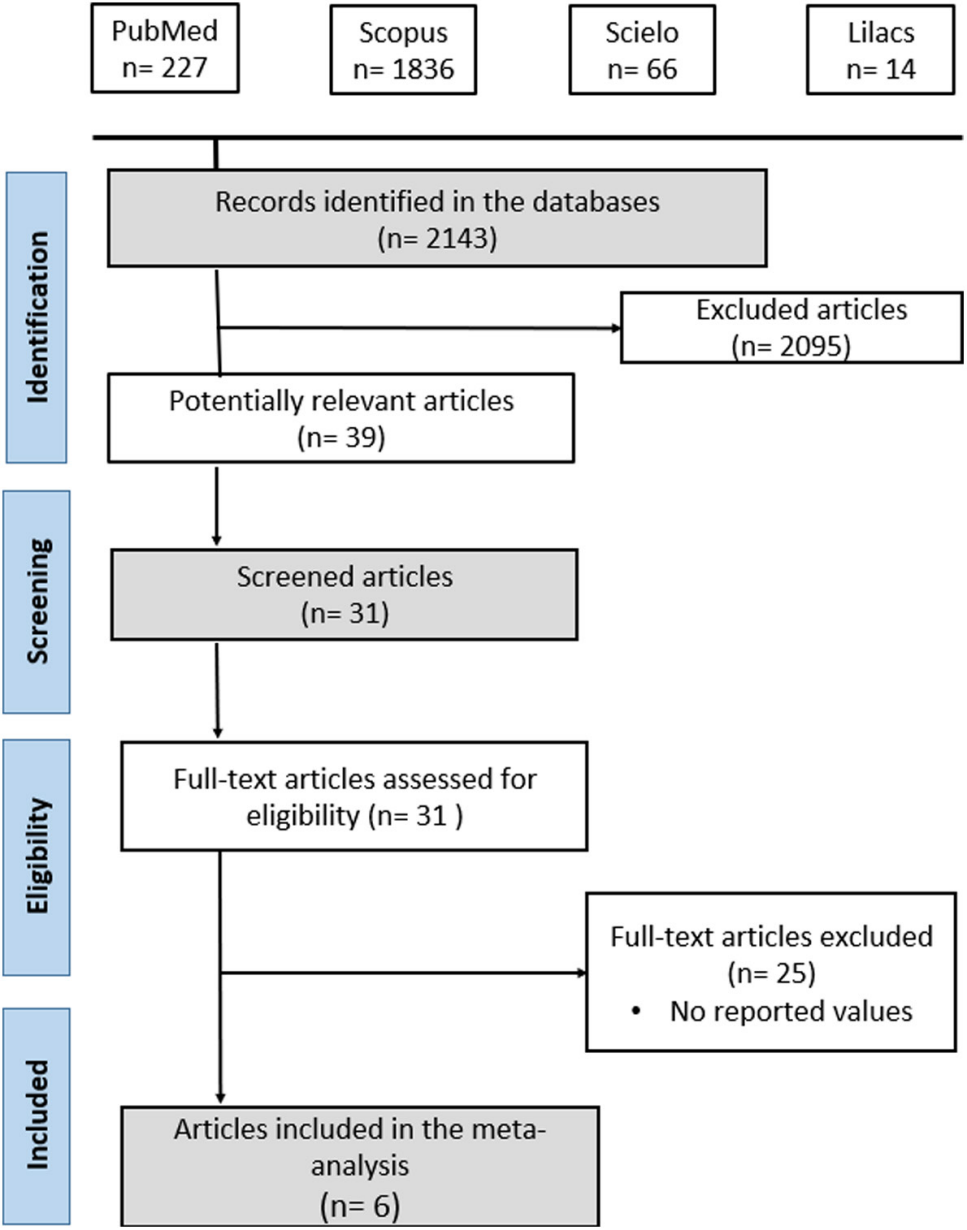
PRISMA 2020 flow diagram.

Figure 2 depicts the flowchart, adhering to the criteria outlined in PRISMA 2020, and illustrates all included studies. Following the completion of the search strategy in the selected bibliographic databases, a total of 2143 scientific articles were initially identified. Subsequently, over 2000 studies were excluded, narrowing it down to 39 potentially relevant articles. By applying screening and eligibility criteria, we arrived at a final sample of six articles, which reported a total of 11 DL models. This facilitated the execution of the corresponding analyses for this meta-analysis.

### Studies’ features

A total of six articles were gathered from five different countries, with two originating from Taiwan and one each from Chile, South Korea, Poland, and Switzerland. These selected articles featured participants of diverse genders and ages, providing insights into 11 algorithms that report diagnostic metrics, including AlexNet, ATASM, DenseNet, CNN-2, Inception-V3, nnU-Net, ResNet, ResNet50, VGG16, VGG-19, and Xception.

The processing involved 165 232 images for diagnosing tendon-related pathologies, with four studies utilizing MRI analysis and two employing soft tissue ultrasounds. Assessments of tendon integrity, tendon nodules, tendon rupture, tendon tear, peritendon, and musculotendinous fat infiltration were emphasized among the tendon-related conditions, with no repetition observed. For a detailed description of each selected article, along with their respective algorithms and metrics, refer to Table 2 (31, 32, 33, 34, 35, 36).

**Table 2:** Characteristics of the included studies.

References	Country	Images, *n*	Imaging	Condition	Algorithm	TP	FP	FN	TN	ACC	SE	SP	AUC
Cho *et al.* (31)	Korea	580	MRI	Tendon integrity	A: VGG16	45	56	11	167	0.76	0.80	0.74	0.83
					B: DenseNet	46	16	10	207	0.91	0.84	0.93	0.92
					C: Xception	51	24	11	193	0.87	0.84	0.89	0.91
Chuang *et al.* (32)	Taiwan	74	US	Nodule tendon	ATASM	173	17	37	193	0.87	0.82	0.91	–
Hess *et al.* (33)	Switzerland	171	MRI	Tendon tear	nnU- Net	2	2	0	56	–	1.0	0.97	–
Kapiński *et al.* (34)	Poland	160000	MRI	Tendon rupture	A: AlexNet	7680	201	309	7787	0.96	0.96	0.97	–
					B: ResNet	7623	44	366	7944	0.97	0.95	0.99	
Lin *et al.* (35)	Taiwan	3801	US	Peritendinous	CNN-2	72	7	3	18	0.74	0.96	0.72	–
Saavedra *et al.* (36)	Chile	606	MRI	Fatty infiltration	A: VGG-19	108	39	6	1512	0.97	0.94	0.97	0.99
					B: ResNet50	105	31	9	1520	0.97	0.92	0.98	0.99
					C: Inception-V3	99	29	15	1522	0.97	0.86	0.98	0.99

### Risk of bias

**Figure 3:**
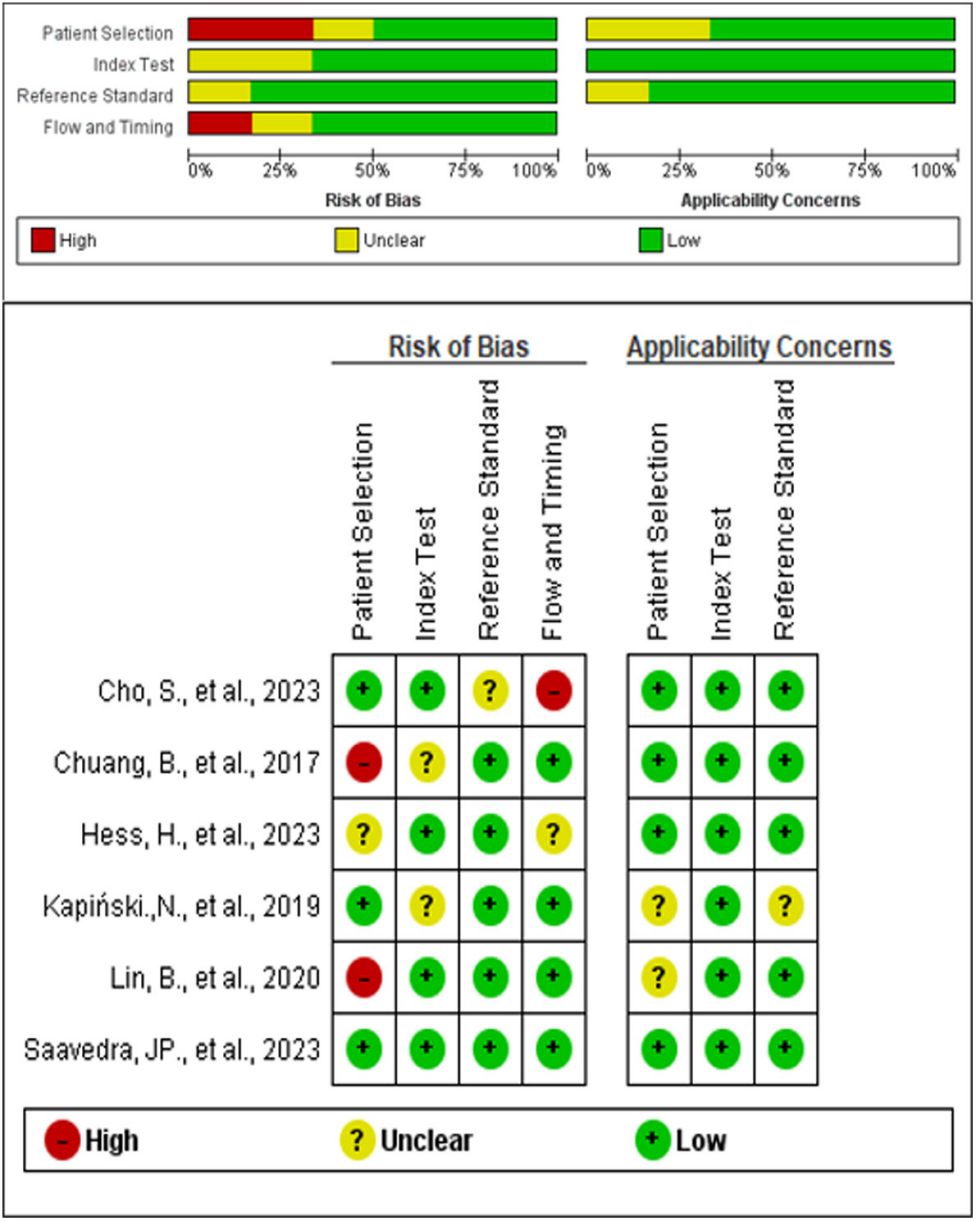
Quality assessment of included studies (31, 32, 33, 34, 35, 36).

The six selected articles underwent scrutiny using the QUADAS-2 methodological tool. Among them, three articles displayed a high risk of bias—one in the dimension of flow and timing, and the other two in the patient selection dimension. In contrast, only one article attained a low risk across all assessed items. Furthermore, five articles featured at least one aspect with unclear information. For a more nuanced understanding, kindly refer to Fig. 3.

### Univariate analysis

**Figure 4:**
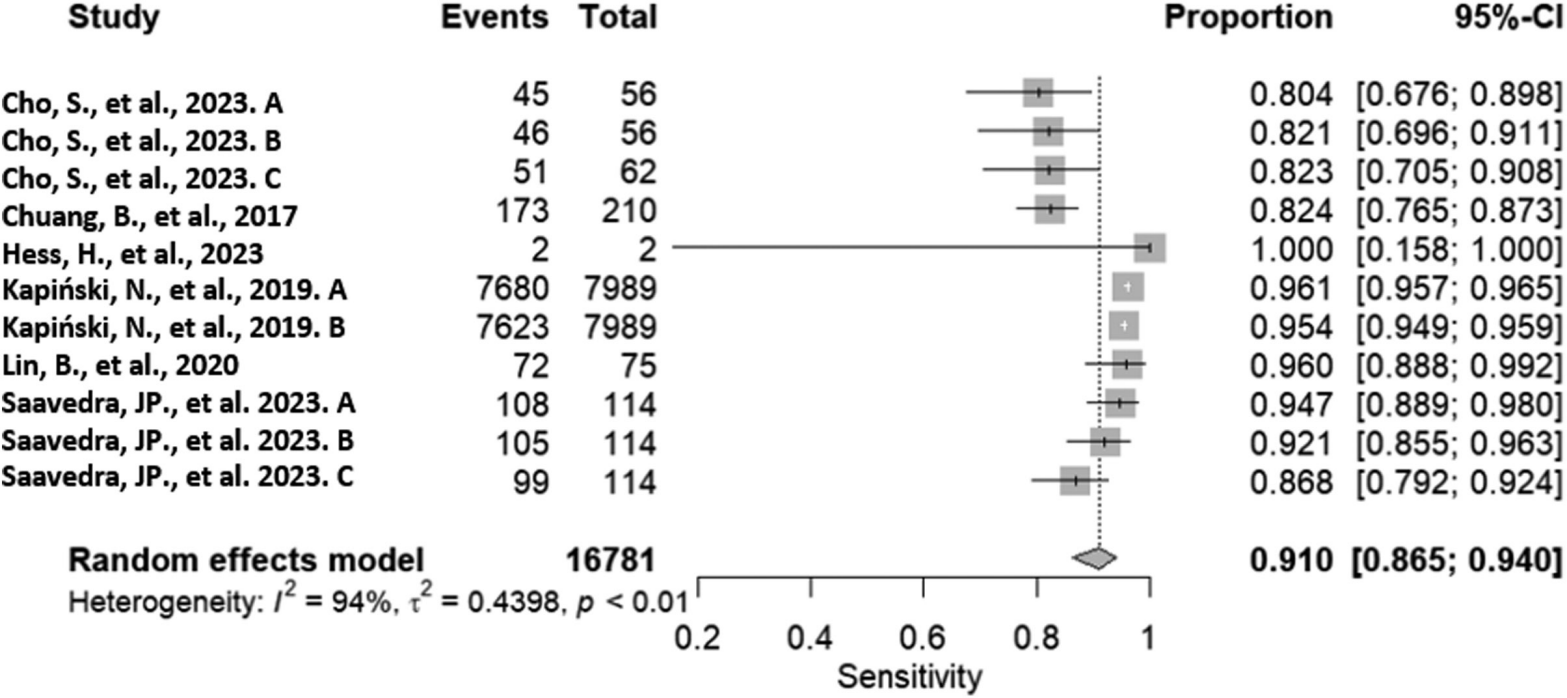
The forest plot of pooled sensitivity (31, 32, 33, 34, 35, 36).

The analysis considered the six included articles, which provided information about 11 algorithms to assess the diagnostic performance of conditions involving tendons using two types of medical images. The random-effects model obtained a sensitivity of 0.910 (95% CI: 0.865; 0.940), demonstrating a high level of accuracy in correctly identifying positive cases. In other words, this means that the evaluated algorithms are capable of effectively identifying positive cases in 91% of instances. However, there is a substantial and significant amount of heterogeneity among the studies included in the analysis, indicated by *τ*²= 0.439, *I*² = 93.6% (90.4%; 95.7%), *P* < 0.0001. Details and graphical representation can be found in Fig. 4.

**Figure 5:**
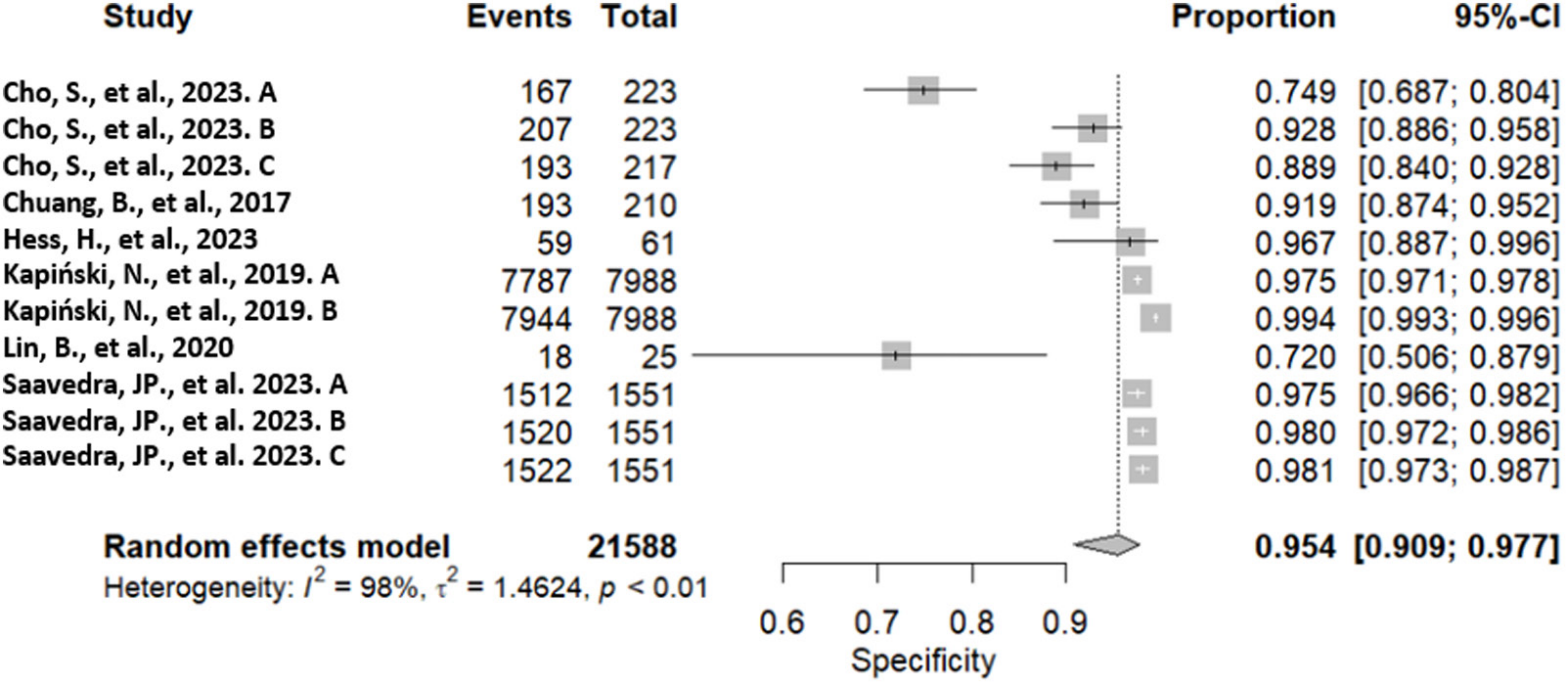
The forest plot of pooled specificity (31, 32, 33, 34, 35, 36).

The random-effects model obtained a specificity value of 0.954 (0.909; 0.977), once again indicating high precision in correctly identifying negative cases. In other words, the algorithms can correctly identify negative cases in 95% of instances or have an estimation error of only 5%. However, specificity exhibits very high and significant heterogeneity in this instance. The reported values are *τ*²= 1.462, *I*² = 98% (97.3%; 98.5%), *P* < 0.0001. Figure 5 provides further graphical details.

The estimated positive likelihood ratio (PLR) was 37.075 (95% CI: 4.654; 69.496). This indicates that the probability of obtaining a positive result in individuals with the studied condition is 37 times higher than in those without the condition. Although the confidence interval reflects some variability in the estimation, the magnitude of the PLR is substantial. Consequently, the test exhibits strong discriminative power to identify the condition of interest.

The estimated negative likelihood ratio (NLR) is 0.114 (95% CI: 0.056; 0.171). This indicates that the probability of obtaining a negative result in individuals with the studied condition is 0.114 times lower than in those without the condition. In other words, the test demonstrates a robust ability to rule out the condition when the result is negative. Although the confidence interval shows some variability in the estimation, the magnitude of the NLR is low, supporting confidence in this assessment. Therefore, the results suggest that the test is effective in excluding the presence of the condition in subjects with a negative result, and this finding has a solid foundation of confidence.

**Figure 6:**
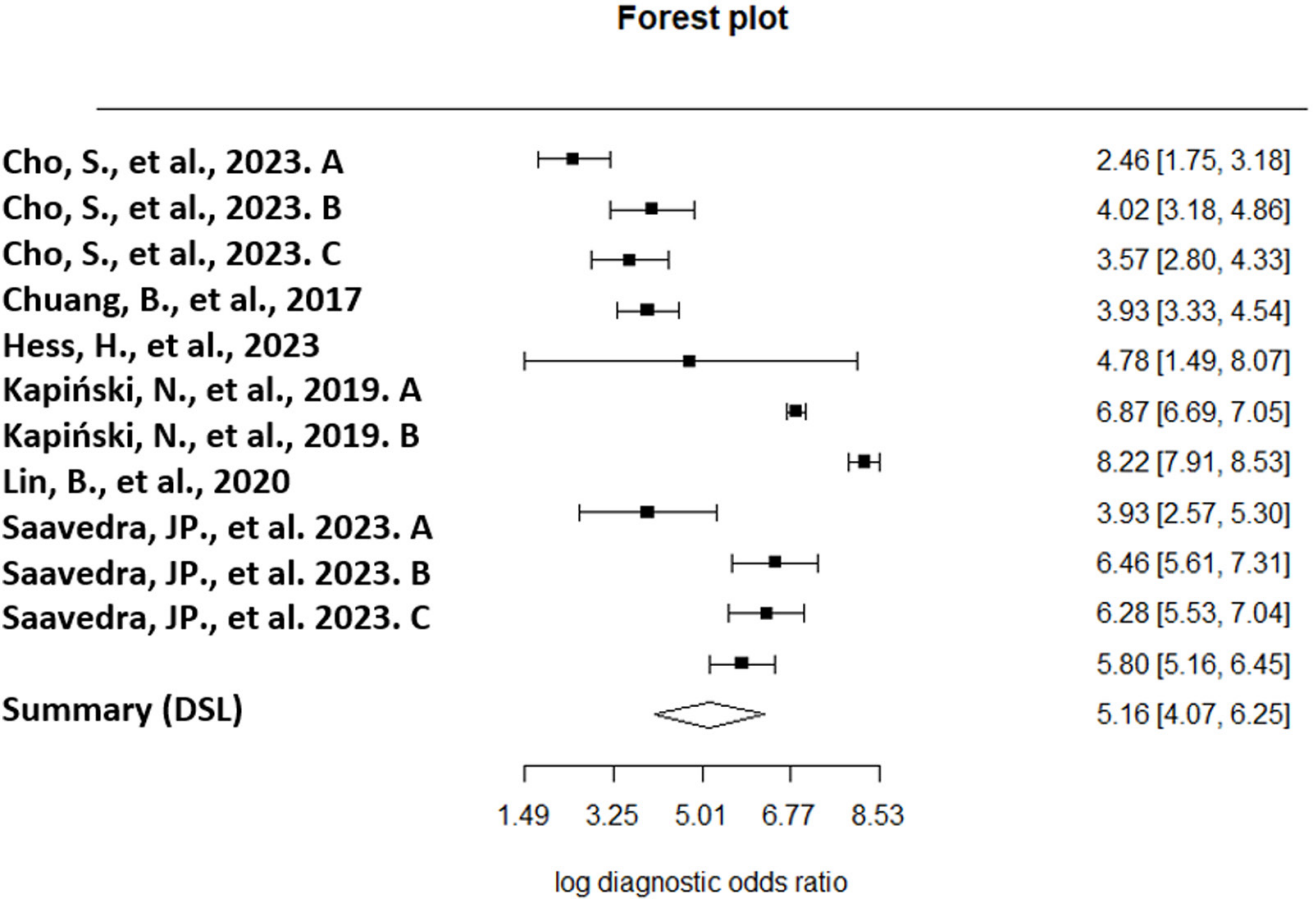
Forest plot of the log diagnostic odds ratios (31, 32, 33, 34, 35, 36).

The diagnostic odds ratio (DOR) was calculated to be 5.220 (95% CI: 4.147; 6.293) with a *P*-value < 0.001. The delivered value suggests a moderately high performance, 5.2 times greater in individuals using the test in the context of diagnostic tests. The lnDOR, on the other hand, presented a slightly lower value of 5.160 (95% CI: 4.070; 6.250) with a (*P* < 0.001). Figure 6 provides further details.

### Bivariate analysis

The random-effects model provided an overall estimate of the diagnostic odds ratio at 184.9695 (95% CI: 63.2688; 540.7679), *P* < 0.0001. Consequently, a robust and significant association exists between the designated categories. Additionally, the value of *τ*²=3.001 (95% CI: 1.3234; 8.9342) suggests a substantial amount of variability in the performance across studies. Furthermore, the *I* ^2^ value of 97.6% (96.7%; 98.2%) suggests that heterogeneity accounts for a substantial proportion of the observed variability, indicating significant discrepancies among the studies.

**Figure 7:**
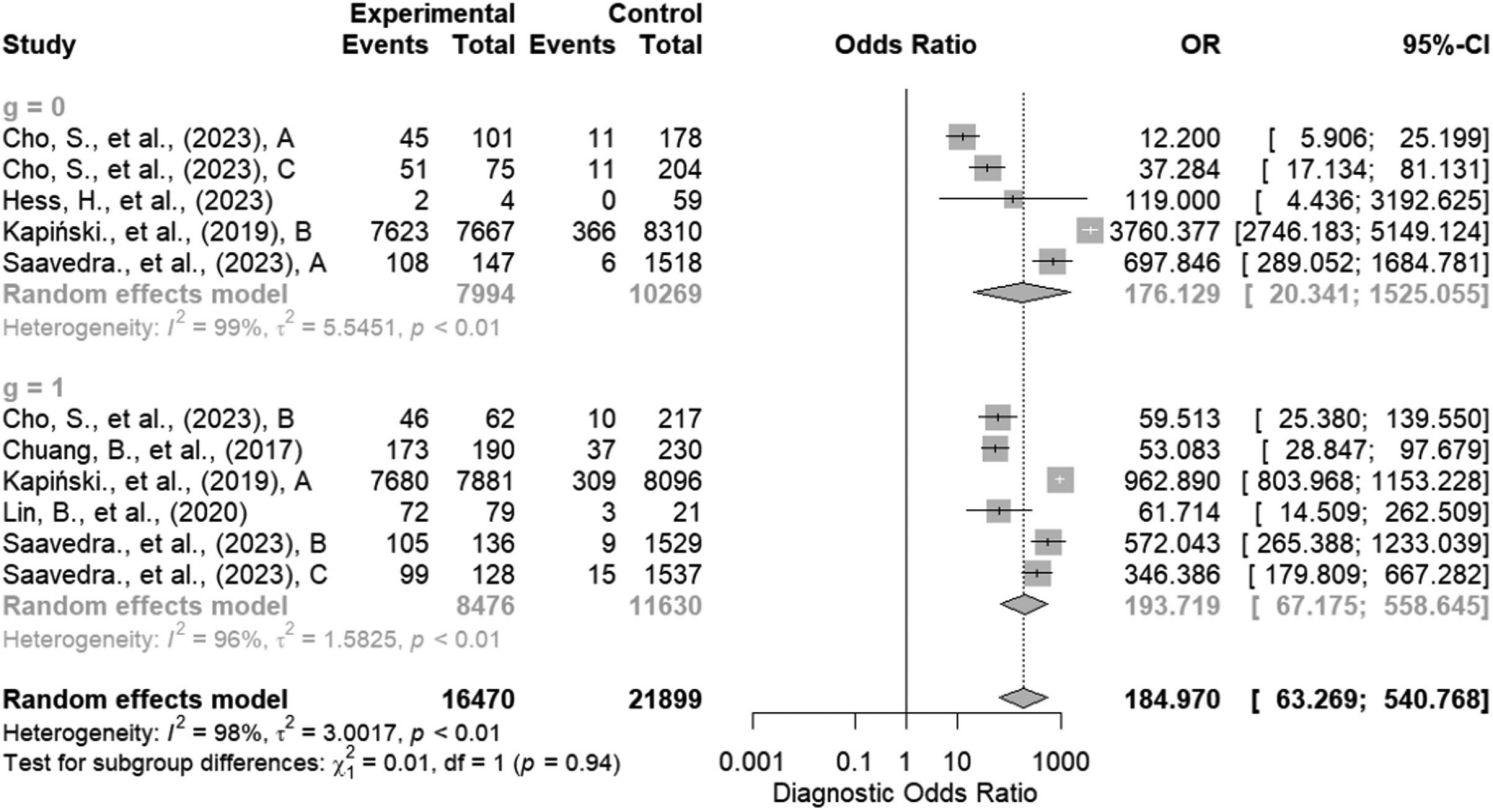
Forest plot of the odds ratios (31, 32, 33, 34, 35, 36).

However, when categorizing algorithms based on their architectural complexity, the category zero, representing those of lower complexity, exhibited an OR = 176.129 (95% CI: 20.341; 1525.055) in the random-effects model, with a *τ*²=5.5451 and *I* ^2^ = 98.6%. On the other hand, the higher complexity group presented an OR = 193.7189 (95% CI: 67.1750; 558.6451) with a *τ*²=1.5825 and *I* ^2^ = 96.1%. The test for differences between the two subgroups yielded a *P*-value of 0.938, suggesting insufficient evidence to assert significant differences in odds ratios between the two subgroups. Additional information is available in Fig. 7.

**Figure 8:**
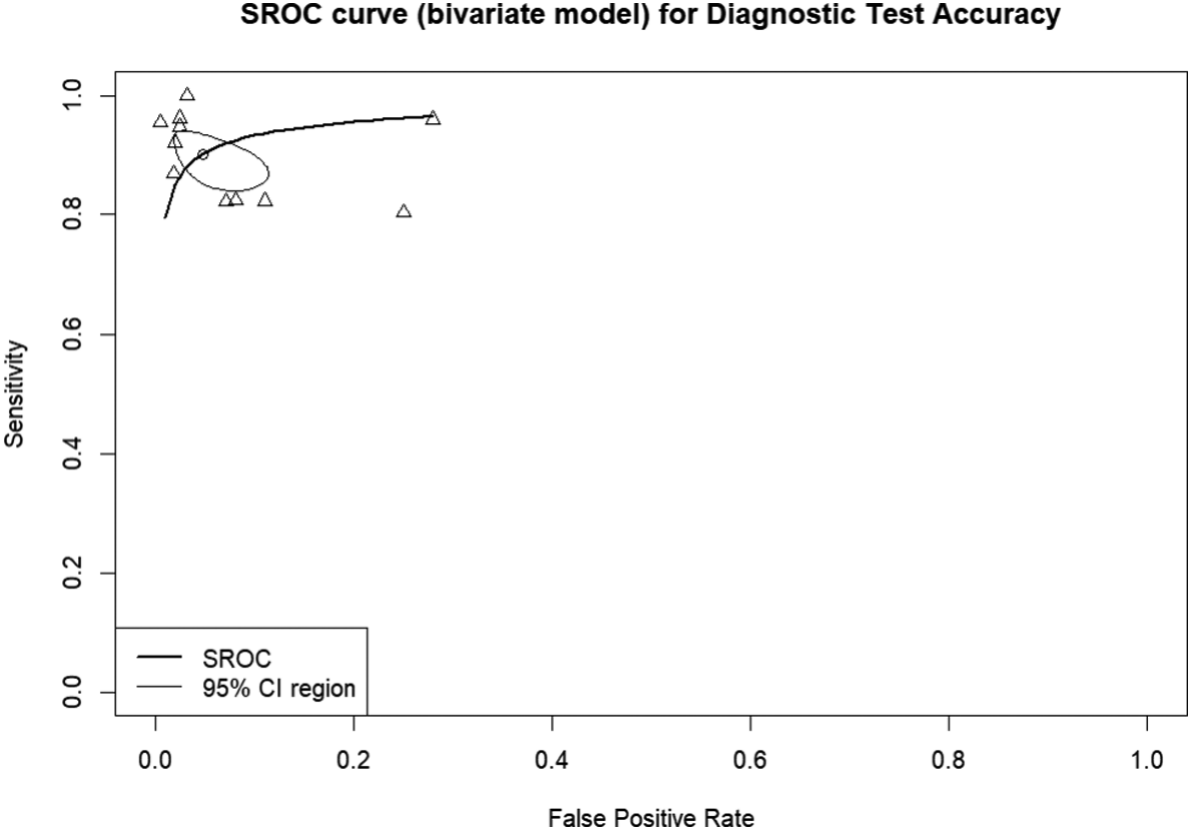
SROC curve (bivariate model) for diagnostic test accuracy.

Finally, the performance of the classification model, as assessed by the AUC estimator, yielded a value of 96%, suggesting a strong overall discriminative power. In other words, there is a very high probability of correct classification. Figure 8 provides a graphical representation of the performance of the evaluated algorithms.

## Discussion

The growing popularity and widespread acceptance of neural networks in the field of medical image recognition within the radiological community can be attributed primarily to their capacity to enhance decision-making for healthcare professionals. This, in turn, significantly improves the efficiency and precision of disease diagnosis and treatment. Both the clinical and scientific communities widely embrace the concept of computer-aided diagnosis (37). This trend is, to a large extent, fueled by advancements in hardware and software technologies, increased availability and access to data, as well as the ongoing enhancement of analysis models (38). Within this context, one might posit that incorporating algorithms with more intricate architectures and a greater number of layers could potentially yield improved performance metrics. However, it's noteworthy that many of these structures share similar modules and mathematical formulations, which could contribute to a more uniform performance across different models (39).

First, this meta-analysis has unprecedentedly highlighted the high capacity of deep learning algorithms to recognize and classify tendinopathy-related alterations with exceptional precision. Furthermore, neural networks demonstrate excellent performance regardless of their specific structure. This behavior reaffirms the versatility of this technological tool and suggests its potential for more frequent use in clinical practice to consistently support the diagnosis of musculoskeletal disorders, particularly those affecting tendons.

In this context, a recent literature review explored the terms ‘orthopedic’, ‘artificial intelligence’, ‘deep learning’, ‘machine learning’, and ‘convolutional neural network’, all of which are keywords incorporated in this article. The review consistently showed an exponential increase in publication records per year, indicating the growing interest among clinicians in utilizing these technologies in their professional practice (40). Additionally, there is a consensus among professionals regarding the advantages associated with the specific use of diagnostic strategies employing image recognition in this discipline (41).

On the other hand, we highlight the vast diversity of existing deep learning techniques, surpassing even other strategies from machine learning for image segmentation, feature selection and extraction, pattern recognition, and classification. DL models enable machines to achieve higher precision due to advancements in methods for analyzing images, thanks to their complex architectures (42). In particular, this is because the layers of a neural network work together as a hierarchical processing system, with each layer taking on a specific role in abstracting features from visual data. This makes it possible to classify things quickly by learning complicated patterns (4).

Furthermore, this article analyzed several of the most popular deep learning architectures for image processing, such as AlexNet, VGG, and ResNet, which demonstrated excellent performance metrics. This has allowed deep learning to become an increasingly utilized tool by specialists today. However, it has a history that goes back to the 1940s with Shallow Neural Networks. This shows that its uses and subsequent changes have made it possible for new, more complex analysis tools to appear every day for the successful processing of medical images in diagnostic support (10).

This ongoing period of expansion is not without challenges and complications, as it requires a plentiful amount of labeled data to properly train a network (43). The difficulty associated with data collection and image labeling may introduce some subjectivity or variability among observers, potentially impacting the accuracy and reliability of the models (44). Another significant challenge pertains to the distribution of data in training and validation sets, which could result in poor external validation when analyzing new or untrained data in the model. Therefore, it is crucial to consider a wide range of clinical conditions during the process to ensure the model's efficiency in all possible scenarios (45). It is highly desirable for clinicians to have a certain level of training and understanding of how different models function to initially comprehend the database's behavior and then decide which structure might best suit the specific requirements of the proposed problem (46).

This meta-analysis also highlights some methodological challenges when reporting diagnostic metrics for medical images. For instance, there is a need for a checklist system to guide researchers on how to conduct diagnostic studies evaluating images in the musculoskeletal area. However, other research groups in the field of dermatology have introduced an innovative checklist for assessing artificial intelligence-based image reports (47). Recently, researchers proposed the checklist guide for AI in Medical Imaging (CLAIM) to ensure compliance with a 42-item checklist based on the 2015 Standards for Reporting Diagnostic Accuracy Studies (STARD). The checklist guide for AI in Medical Imaging (CLAIM) is designed to evaluate the integrity of specific reports for AI applications in medical images. However, since its publication, adherence to these requirements has not been clear (48). For this reason, we believe that researchers should, at the very least, report basic diagnostic performance metrics, incorporating detailed information on TP, FN, FP, and TN. Furthermore, articles should consistently report the use of multiple DL algorithms for the same issue, as we observed varying performance capabilities.

Finally, this present study has some limitations, including the low number of selected articles. However, regardless of the quantity of articles obtained, the crucial aspect is that in future meta-analyses, the focus should be on evaluating the reported models rather than the quantity of articles found. Strict adherence to a research question and a search strategy consistent with the results explains this outcome, indicating that even in the musculoskeletal diagnostic field, with a pathology as prevalent as tendon issues, there is still room for the development of these strategies. Moreover, the evaluation of different tendon disorders using various algorithms did not facilitate a comparison of similar variables that would allow for an assessment of a unified diagnostic experience. In this regard, there is a need for more studies replicating similar diagnostic strategies to obtain a genuine evaluation of the diagnostic capabilities of these tools.

When contemplating the future use of deep learning to identify tendon issues in medical images, it's important to note that as models and algorithms continue to improve, they will enhance their performance every day. This will make it easier for humans and machines to collaborate and learn. Therefore, we firmly believe that transdisciplinary efforts should incorporate the use of technology and advanced computational analysis. Regarding the attainment of more efficient models, improvement will occur as healthcare providers increasingly adopt them through the effective implementation of diagnostic support systems that facilitate real-time decision-making. On the other hand, this group of researchers understands that these applications in detecting tendon anomalies are relatively recent. Still, as their use becomes more commonplace, predictive analyses will anticipate the development of actual pathology. Implementing appropriate preventive clinical measures will extend the lifespan of this biological tissue.

## Conclusions

The performance of deep learning models in diagnosing tendon-related disorders has been exceptional, showcasing high diagnostic precision irrespective of model complexity. We encourage researchers to persist in utilizing these tools, employing multiple models concurrently, and providing detailed results to enhance metrics. However, challenges persist, requiring clinicians to enhance their understanding of AI concepts and integrate AI into interdisciplinary teams. Strategic solutions include developing consensus guidelines for reporting diagnostic metrics, collaborating with engineering professionals for the digital transition in healthcare, and improving interoperability in electronic medical record systems. Moreover, there is a need to develop hybrid algorithms to enhance accuracy and speed in identifying tendon problems.

The present research has some limitations, such as the lack of inclusion of other very common soft tissue structures for image analysis, such as ligaments. Additionally, only architectures such as DL, CNN, and ML models were considered, but not other more traditional ones such as regressions or KNN, or some more current ones such as agent models. Finally, this article focuses on the classification capacity of the models rather than segmentation, leaving an interesting space for future research lines.

## ICMJE Conflict of Interest Statement

The authors declare that there is no conflict of interest that could be perceived as prejudicing the impartiality of the study reported.

## Funding Statement

This study did not receive any specific grant from any funding agency in the public, commercial, or not-for-profit sector.

## Author contribution statement

Conceptualization: GD; software and statistical analysis: GD; data curation: GD, CR, DS; writing-original draft preparation: GD; writing-review: CJ and FF; supervision and editing: FF. All authors have read and agreed to the published version of the manuscript.

## References

[1] Raja Santhi A & Muthuswamy P. Industry 5.0 or industry 4.0S? Introduction to industry 4.0 and a peek into the prospective industry 5.0 technologies. International Journal on Interactive Design and Manufacturing (IJIDeM) 2023 17 947–979. (10.1007/s12008-023-01217-8) PMC9899508 40478102

[2] Kern C Gerdon F Bach RL Keusch F & Kreuter F. Humans versus machines: who is perceived to decide fairer? Experimental evidence on attitudes toward automated decision-making. Patterns 2022 3 100591. (10.1016/j.patter.2022.100591) 36277823 PMC9583126

[3] Olveres J González G Torres F Moreno-Tagle JC Carbajal-Degante E Valencia-Rodríguez A Méndez-Sánchez N & Escalante-Ramírez B. What is new in computer vision and artificial intelligence in medical image analysis applications. Quantitative Imaging in Medicine and Surgery 2021 11 3830–3853. (10.21037/qims-20-1151) 34341753 PMC8245941

[4] Rana M & Bhushan M. Machine learning and deep learning approach for medical image analysis: diagnosis to detection. Multimedia Tools and Applications 2023 82 26731–26769.(10.1007/s11042-022-14305-w) PMC9788870 36588765

[5] De MR Gang GJ Li X & Wang G. Comparison of deep learning and human observer performance for detection and characterization of simulated lesions. Journal of Medical Imaging 6 2019 025503. (10.1117/1JMI62025503) 31263738 PMC6586983

[6] Jin D Harrison AP Zhang L Yan K Wang Y Cai J, *et al.* Artificial intelligence in radiology. Artificial Intelligence in Medicine 2021 265–289.(10.1016/FB978-0-12-821259-2.00014-4)

[7] Alzubaidi L Zhang J Humaidi AJ Al-Dujaili A Duan Y Al-Shamma O, *et al.* Review of deep learning: concepts, CNN architectures, challenges, applications, future directions. Journal of Big Data 2021 81 1–74. (10.1186/s40537-021-00444-8) PMC8010506 33816053

[8] Hosny A Parmar C Quackenbush J Schwartz LH & Aerts HJWL. Artificial intelligence in radiology. Nature Reviews. Cancer 2018 18 500–510. (10.1038/s41568-018-0016-5) 29777175 PMC6268174

[9] Davenport T & Kalakota R. The potential for artificial intelligence in healthcare. Future Healthcare Journal 2019 6 94–98. (10.7861/futurehosp.6-2-94) PMC6616181 31363513

[10] Suganyadevi S Seethalakshmi V & Balasamy K. A review on deep learning in medical image analysis. International Journal of Multimedia Information Retrieval 2022 11 19–38 (10.1007/s13735-021-00218-1) 34513553 PMC8417661

[11] Lecun Y Bengio Y & Hinton G. Deep learning. Nature 2015 521 436–444. (10.1038/nature14539) 26017442

[12] Sarker IH. Deep learning: a comprehensive overview on techniques, taxonomy, applications and research directions. SN Computer Science 2021 2 420. (10.1007/s42979-021-00815-1) 34426802 PMC8372231

[13] Miotto R Wang F Wang S Jiang X & Dudley JT. Deep learning for healthcare: review, opportunities and challenges. Briefings in Bioinformatics 2018 19 1236–1246 (10.1093/bib/bbx044) 28481991 PMC6455466

[14] Nossier SA Wall J Moniri M Glackin C & Cannings N. An experimental analysis of deep learning architectures for supervised speech enhancement. Electronics 2020 10 17. (10.3390/electronics10010017)

[15] Ahmed SF Alam MSB Hassan M Rozbu MR Ishtiak T Rafa N Mofijur M Shawkat Ali ABM & Gandomi AH. Deep learning modelling techniques: current progress, applications, advantages, and challenges. Artificial Intelligence Review 2023 56 13521–13617. (10.1007/s10462-023-10466-8)

[16] Nuzzi R Boscia G Marolo P & Ricardi F. The impact of artificial intelligence and deep learning in eye diseases: a review. Frontiers in Medicine 2021 8 710329. (10.3389/fmed.2021.710329) 34527682 PMC8437147

[17] Bhat M Rabindranath M Chara BS & Simonetto DA. Artificial intelligence, machine learning, and deep learning in liver transplantation. Journal of Hepatology 2023 78 1216–1233. (10.1016/j.jhep.2023.01.006) 37208107

[18] Choy SP Kim BJ Paolino A Tan WR Lim SML Seo J Tan SP Francis L Tsakok T Simpson M, *et al.* Systematic review of deep learning image analyses for the diagnosis and monitoring of skin disease. npj Digital Medicine 2023 6 180. (10.1038/s41746-023-00914-8) 37758829 PMC10533565

[19] Belciug S. Learning deep neural networks’ architectures using differential evolution. Case study: medical imaging processing. Computers in Biology and Medicine 2022 146 105623. (10.1016/j.compbiomed.2022.105623) 35751202 PMC9112664

[20] Simonyan K & Zisserman A. Very deep convolutional networks for large-scale image recognition. arXiv 1409.1556. (10.48550/arXiv.1409.1556)

[21] Szegedy C Vanhoucke V Ioffe S Shlens J & Wojna Z. Rethinking the inception architecture for computer vision. 2016 IEEE Conference on Computer Vision and Pattern Recognition (CVPR) 2016, pp. 2818–2826. (10.1109/CVPR.2016.308)

[22] Huang G Liu Z Van Der Maaten L & Weinberger KQ. Densely connected convolutional networks 2017 IEEE Conference on Computer Vision and Pattern Recognition (CVPR) 2017, pp. 2261–2269 . (10.1109/CVPR.2017.243)

[23] He K Zhang X Ren S & Sun J. Deep residual learning for image recognition 2016 IEEE Conference on Computer Vision and Pattern Recognition (CVPR) 2016, pp. 770–778. (10.1109/CVPR.2016.90)

[24] Federer SJ & Jones GG. Artificial intelligence in orthopaedics: a scoping review. PLoS One 2021 16 e0260471 (10.1371/journal.pone.0260471) 34813611 PMC8610245

[25] Lalehzarian SP Gowd AK & Liu JN. Machine learning in orthopaedic surgery. World Journal of Orthopedics 2021 12 685–699. (10.5312/wjo.v12.i9.685) 34631452 PMC8472446

[26] Droppelmann G Feijoo F Greene C Tello M Rosales J Yáñez R Jorquera C & Prieto D. Ultrasound findings in lateral elbow tendinopathy: a retrospective analysis of radiological tendon features. F1000Research 2022 11 44. (10.12688/f1000research.73441.1)

[27] Droppelmann G Tello M García N Greene C Jorquera C & Feijoo F. Lateral elbow tendinopathy and artificial intelligence: binary and multilabel findings detection using machine learning algorithms. Frontiers in Medicine 2022 9 945698. (10.3389/fmed.2022.945698) 36213676 PMC9537568

[28] Whiting P. QUADAS-2 | Bristol Medical School: Population Health Sciences. University of Bristol 2011. Available at: https://www.bristol.ac.uk/population-health-sciences/projects/quadas/quadas-2/.

[29] Shim SR Kim SJ & Lee J. Diagnostic test accuracy: application and practice using R software. Epidemiology and Health 2019 41 1–8. (10.4178/epih.e2019007) PMC6545496 30999739

[30] Shim SR. Meta-analysis of diagnostic test accuracy studies with multiple thresholds for data integration. Epidemiology and Health 2022 44 e2022083. (10.4178/epih.e2022083) 36228672 PMC10106543

[31] Cho SH & Kim YS. Prediction of Retear after arthroscopic rotator cuff repair based on intraoperative arthroscopic images using deep learning. American Journal of Sports Medicine 2023 51 2824–2830. (10.1177/03635465231189201) 37565449

[32] Chuang BI Kuo LC Yang TH Su FC Jou IM Lin WJ & Sun YN. A medical imaging analysis system for trigger finger using an adaptive texture-based active shape model (ATASM) in ultrasound images. PLOS ONE 2017 12 e0187042. (10.1371/journal.pone.0187042) 29077737 PMC5659776

[33] Hess H Ruckli AC Bürki F Gerber N Menzemer J Burger J Schär M Zumstein MA & Gerber K. Deep-learning-based segmentation of the shoulder from MRI with inference accuracy prediction. Diagnostics 2023 13 1–13. (10.3390/diagnostics13101668) PMC10217676 37238157

[34] Kapiński N Zieliński J Borucki BA Trzciński T Ciszkowska-Łysoń B Zdanowicz U Śmigielski R & Nowiński KS. Monitoring of the Achilles tendon healing process: can artificial intelligence be helpful? Acta of Bioengineering and Biomechanics 2019 21 103–111. 31197280

[35] Lin BS Chen JL Tu YH Shih YX Lin YC Chi WL & Wu YC. Using deep learning in ultrasound imaging of bicipital peritendinous effusion to grade inflammation severity. IEEE Journal of Biomedical and Health Informatics 2020 24 1037–1045. (10.1109/JBHI.2020.2968815) 31985446

[36] Saavedra JP Droppelmann G García N Jorquera C & Feijoo F. High-accuracy detection of supraspinatus fatty infiltration in shoulder MRI using convolutional neural network algorithms. Frontiers in Medicine 2023 10 1070499. (10.3389/fmed.2023.1070499) 37305126 PMC10248442

[37] Chan HP Samala RK Hadjiiski LM & Zhou C. Deep learning in medical image analysis. Advances in Experimental Medicine and Biology 2020 1213 3–21.(10.1007/978-3-030-33128-3_1) 32030660 PMC7442218

[38] Bohr A & Memarzadeh K. The rise of artificial intelligence in healthcare applications. Artificial Intelligence in Healthcare 2020 25–60. (10.1016/B978-0-12-818438-7.00002-2)

[39] Vakalopoulou M Christodoulidis S Burgos N Colliot O & Lepetit V. Basics and convolutional neural networks (CNNs). Machine Learning for Brain Disorders 2023. Available at: 10.1007/978-1-0716-3195-9_3. 37988533

[40] Farhadi F Barnes MR Sugito HR Sin JM Levy HE. Applications of artificial intelligence in orthopaedic surgery. Frontiers in Medical Technology 2022 4 995526. (10.3389/fmedt.2022.995526) 36590152 PMC9797865

[41] Myers TG Ramkumar PN Ricciardi BF Urish KL Kipper J & Ketonis C. Artificial intelligence and orthopaedics: an introduction for clinicians. Journal of Bone and Joint Surgery 2020 102 830–840. (10.2106/JBJS.19.01128) PMC7508289 32379124

[42] Sistaninejhad B Rasi H & Nayeri P. A review paper about deep learning for medical image analysis. Computational and Mathematical Methods in Medicine 2023 2023 7091301 (10.1155/2023/7091301) 37284172 PMC10241570

[43] Li M Jiang Y Zhang Y & Zhu H. Medical image analysis using deep learning algorithms. Frontiers in Public Health 2023 11 1273253. (10.3389/fpubh.2023.1273253) 38026291 PMC10662291

[44] Ghassemi M Naumann T Schulam P Beam AL Chen IY & Ranganath R. A review of challenges and opportunities in machine learning for health. AMIA Joint Summits on Translational Science Proceedings. 2020 2020 191–200. 32477638 PMC7233077

[45] Xu Y & Goodacre R. On splitting training and validation set: a comparative study of cross-validation, bootstrap and systematic sampling for estimating the generalization performance of supervised learning. Journal of Analysis and Testing 2018 2 249–262. (10.1007/s41664-018-0068-2) 30842888 PMC6373628

[46] Chen D Liu S Kingsbury P Sohn S Storlie CB Habermann EB, *et al.* Deep learning and alternative learning strategies for retrospective real-world clinical data. NPJ Digital Medicine 2019 21 1–5.(10.1038/s41746-019-0122-0) PMC6550223 31304389

[47] Daneshjou R Barata C Betz-Stablein B Celebi ME Codella N Combalia M Guitera P Gutman D Halpern A Helba B, *et al.* Checklist for evaluation of image-based artificial intelligence reports in dermatology: clear derm consensus guidelines from the international skin imaging collaboration artificial intelligence working group. JAMA Dermatology 2022 158 90–96. (10.1001/jamadermatol.2021.4915) 34851366 PMC9845064

[48] Sivanesan U Wu K McInnes MDF Dhindsa K Salehi F & van der Pol CB. Checklist for artificial intelligence in medical imaging reporting adherence in peer-reviewed and preprint manuscripts with the highest Altmetric attention scores: a meta-research study. Canadian Association of Radiologists Journal 2023 74 334–342. (10.1177/08465371221134056) 36301600

